# Tumor-Derived Small Extracellular Vesicles Induce Pro-Inflammatory Cytokine Expression and PD-L1 Regulation in M0 Macrophages via IL-6/STAT3 and TLR4 Signaling Pathways

**DOI:** 10.3390/ijms222212118

**Published:** 2021-11-09

**Authors:** Marzia Pucci, Stefania Raimondo, Ornella Urzì, Marta Moschetti, Maria Antonietta Di Bella, Alice Conigliaro, Nadia Caccamo, Marco Pio La Manna, Simona Fontana, Riccardo Alessandro

**Affiliations:** 1 Department of Biomedicine, Neuroscience and Advanced Diagnostics (Bi.N.D), University of Palermo, 90133 Palermo, Italy; marzia.pucci@unipa.it (M.P.); stefania.raimondo@unipa.it (S.R.); ornella.urzi@unipa.it (O.U.); marta.moschetti@unipa.it (M.M.); m.antonietta.dibella@unipa.it (M.A.D.B.); alice.conigliaro@unipa.it (A.C.); nadia.caccamo@unipa.it (N.C.); marcopio.lamanna@unipa.it (M.P.L.M.); riccardo.alessandro@unipa.it (R.A.); 2 Central Laboratory of Advanced Diagnosis and Biomedical Research, 90133 Palermo, Italy; 3 Institute for Biomedical Research and Innovation (IRIB), National Research Council (CNR), 90146 Palermo, Italy

**Keywords:** small extracellular vesicles, M0 macrophages, PD-L1, TLR4, colorectal cancer, multiple myeloma

## Abstract

Tumor-associated macrophages play a key role in promoting tumor progression by exerting an immunosuppressive phenotype associated with the expression of programmed cell death ligand 1 (PD-L1). It is well known that tumor-derived small extracellular vesicles (SEVs) affect the tumor microenvironment, influencing TAM behavior. The present study aimed to examine the effect of SEVs derived from colon cancer and multiple myeloma cells on macrophage functions. Non-polarized macrophages (M0) differentiated from THP-1 cells were co-cultured with SEVs derived from a colorectal cancer (CRC) cell line, SW480, and a multiple myeloma (MM) cell line, MM1.S. The expression of PD-L1, interleukin-6 (IL-6), and other inflammatory cytokines as well as of the underlying molecular mechanisms were evaluated. Our results indicate that SEVs can significantly upregulate the expressions of PD-L1 and IL-6 at both the mRNA and protein levels and can activate the STAT3 signaling pathway. Furthermore, we identified the TLR4/NF-kB pathway as a convergent mechanism for SEV-mediated PD-L1 expression. Overall, these preliminary data suggest that SEVs contribute to the formation of an immunosuppressive microenvironment.

## 1. Introduction

Tumor immunoescape is a well-known hallmark of cancer [1], and it is mainly caused by the activation of the programmed cell-death protein 1 (PD1)/PD-L1 signaling pathway, which inhibits T cell-mediated antitumor responses [2,3,4]. Programmed death ligand 1 (PD-L1 and CD274) is an immunosuppressive protein that, binding directly to its receptor PD-1 on tumor-infiltrating lymphocytes, attenuates T cell activation, induces the apoptosis of activated T cells, as well as induces T-cell anergy and exhaustion [5,6], allowing tumors to evade immune attacks.

Data in the literature reported that PD-L1 is highly expressed on the surface of tumor cells, including melanoma, glioblastoma, lung cancer, renal cancer, gastric cancer, colorectal cancer, pancreatic cancer, breast cancer, and ovarian cancers [7,8,9,10,11,12,13]. Different components of the tumor microenvironment can affect cancer progression, playing a key role in tumor-promoting immunoescape; among them, tumor-associated macrophages as well as tumor-derived small extracellular vesicles (SEVs) represent key players in this process and are currently being explored as therapeutic targets [14,15,16].

Based on the response to different stimuli, macrophages are classified into two distinct subtypes: classically activated/pro-inflammatory (M1) and alternatively activated/reparative (M2) [17,18]. M1-like macrophages kill invading pathogens and tumor cells, while M2-like macrophages play a key role in tissue repair and tumor progression. Tumor-associated macrophages, characterized by M2-like phenotype, represent one of the most important immune cell types in the tumor microenvironment and play a key role in different aspects of cancer progression such as immunosuppression [19]. Recent studies showed that PD-L1 is highly expressed on tumor-associated macrophages, thus suppressing antitumor immune responses [20,21,22,23,24,25,26]. Kubota K. et al. demonstrated that CD163^+^CD204^+^ tumor-associated macrophages promote T-cell apoptosis and immunosuppression via IL-10 and PD-L1, predicting unfavorable prognosis in oral squamous cell carcinoma patients [15].

Recent evidence reported that PD-L1 not only can be expressed on the surface of tumor or immune cells but also can be secreted through extracellular vesicles (EVs).

Extracellular vesicles are biologically active small membrane-bound vesicles secreted by almost all types of cells in biological fluids. EVs represent a new means of communication established by tumor cells and the tumor microenvironment, and several studies have highlighted that they actively participate in tumor progression. Recent evidence has explored the importance of PD-L1 carried by tumor-derived EVs in evading host immune surveillance [27]. Moreover, the role of tumor-derived EVs in modulating PD-L1 expression in target cells has been reported. Haderk et al. showed that chronic lymphocytic leukemia-derived EVs induce the upregulation of PD-L1 in treated monocytes, thus contributing to a tumor-supportive microenvironment in the disease [28]. Kim et al. reported that EVs derived from lung cancer cells contain PD-L1 and are important mediators of tumor immunoescape. These inhibited interferon-γ (IFN-γ) secretion in Jurkat T cells, thus blocking CD8^+^ T-cell activity and inducing their apoptosis [29]. Gabrusiewicz et al. reported for the first time that exosomes released by glioblastoma-derived stem cells can polarize the CD14^+^ monocytes precursor, inducing immunosuppressive M2 macrophage phenotype polarization through the induction of PD-L1; this mechanism is partly mediated by STAT3 activation [30].

In light of the data showing activation of the PD-1/PD-L1 pathway in promoting tumor immunoescape, several anti-PD-1/PD-L1 monoclonal antibodies have been developed. Although the clinical applications of these checkpoint blockades is promising, the successful and long-lasting responses are achieved in only a fraction of patients [31].

Accumulating evidence suggests that the inflammatory cytokines and chemokines play critical roles in cancer immunotherapy [32] and can be responsible for the limited efficacy of the immune-checkpoint blockades. IL-6 is one of the major cytokines released in the tumor microenvironment, and it is involved in multiple processes of tumor development, such as in modeling the immune responses in cancers [33]. Lamano et al. identified glioblastoma-derived IL-6 as a cytokine that is necessary for myeloid PD-L1 induction in Glioblastoma through a STAT3-dependent mechanism [34].

Data in the literature correlated elevated serum levels of IL-6 in colorectal cancer (CRC) patients with advanced tumors and poor prognosis. Jibin Li et al. showed that IL-6 overexpression induces strong immunosuppression in the CRC microenvironment. In particular, they showed that, in CRC mouse models with IL-6 overexpression, the number of CD8^+^ T cells and CD4^+^ T cells decreased. Moreover, they showed that PD-L1 expression was upregulated in the tumors with IL-6 overexpression [35]. Jizhong et al. showed that PD-L1 was expressed in most multiple myeloma (MM) plasma cells and inhibit T-cell functions, representing a possible immunoescape mechanism that could be targeted therapeutically through the inhibition of MyD88/TRAF6 and MEK/ERK/STAT1 [36].

It is well-known that chronic inflammation promotes an inflammatory microenvironment that can in turn support and stimulate tumor progression. Beyond the above mechanisms, several papers reported the nuclear factor kappa-light-chain-enhancer of activated B cells (NF-κB) as a key positive regulator of PD-L1 expression in cancer [37,38,39] with a key role in regulating macrophage function in tumors [40]. Moreover, lipopolysaccharides (LPS) can stimulate PD-L1 expression in macrophages via toll-like receptor (TLR) signaling [41]. Furthermore, melanoma-derived extracellular vesicles carrying heat shock protein (HSP)-86 can stimulate PD-L1 expression in myeloid cells via TLR4 signaling.

In the present study, we aim to examine the effects of small EVs (SEVs) from both colorectal cancer (CRC-SEVs) and multiple myeloma (MM-SEVs) on M0 macrophage (M0-M) functions, in particular on PD-L1 and pro-inflammatory cytokine expression.

## 2. Results

### 2.1. Tumor-Derived SEVs Are Internalized by M0-M

**Figure 1:**
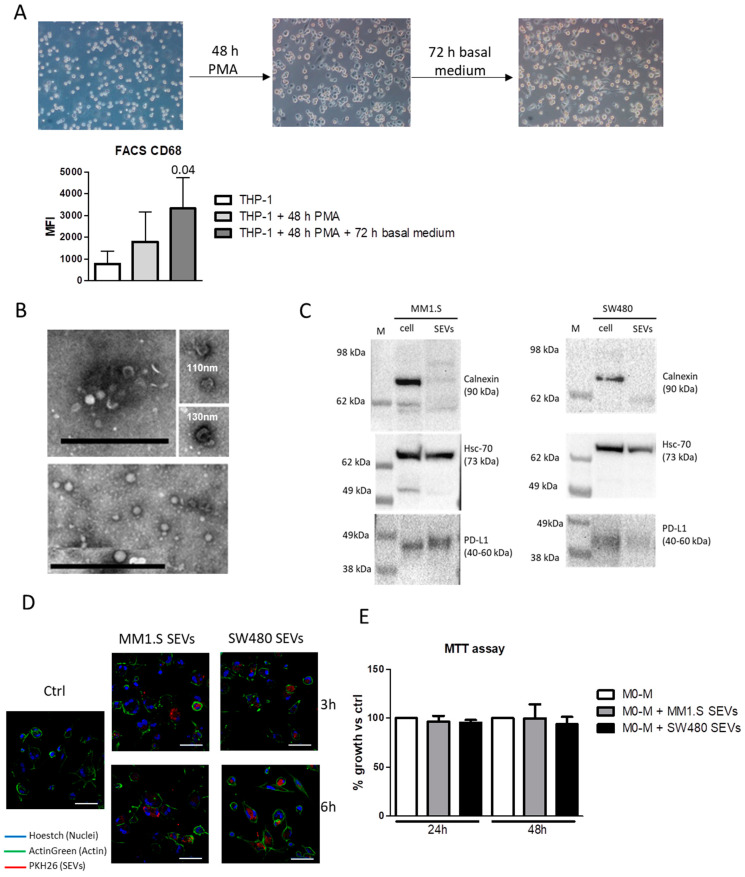
(**A**, upper panel) Representative images of THP-1 monocytes before and after differentiation, acquired with a 10X objective. (**A**, lower panel) FACS analysis of CD68 in THP-1 monocytes before and after differentiation with Phorbol 12-myristate 13-acetate (PMA). The statistical significance of the differences was analyzed using a two-tailed Student’s *t*-test. The reported values are the mean ± standard deviation of three replicates. (**B**) Representative TEM micrographs showing the SEVs isolated from the culture medium of SW480 (upper panel) and MM1.S (lower panel); the insets show details of the single vesicles with their measurements in corresponding images representing the diameter. Scale bar: 1 µm (**C**) Western Blot analysis of Calnexin, HSC-70, and PD-L1 in MM1.S and SW480 cell lysates and the derived SEVs. (**D**) Cellular internalization of MM1.S and SW480-derived SEVs into M0 macrophages (M0-M) was analyzed by confocal microscopy Nikon A1. The median focal plane is reported in the figure. M0-M were incubated for 3 and 6 h with MM1.S and SW480 SEVs labeled with PKH26 (red). M0-M cells were stained with ActinGreen (green), and nuclear counterstaining was performed using Hoescht (blue). (scale bar = 50 µM). (**E**) MTT assay of M0-M treated with MM1.S- and SW480-SEVs for 24 and 48 h. The reported values are the mean ± standard deviation of four replicates.

THP-1 monocytes were differentiated into M0-M as described in the Material and Method section and analyzed by assessing the level of the M0-M marker CD68 which was significantly increased after 5 days of treatment (Figure 1A). The characterization of MM-SEVs and CRC-SEVs was performed using transmission electron microscopy (TEM) and Western Blot analysis. We found that most of the vesicles appeared with well-known cup-shaped morphology and have a diameter ranging from 60 to 130 nm (Figure 1B); SEVs were positive for the typical marker HSP70 but negative for Calnexin, a marker of endoplasmic reticulum (Figure 1C). Interestingly, the immunosuppressive molecule PD-L1 was found in both MM1.S and SW480 cell lysates as well as in the EVs (Figure 1C and Supplementary Figure S1). To investigate the uptake by M0-M, the EVs were labeled with PKH26 and incubated for 3 and 6 h with M0-M. Fluorescent microscopy analysis revealed that, after 3 h, both MM-EVs and SW480-EVs were already internalized and localized inside the cells (Figure 1D). We found that EVs uptake did not affect M0-M viability (Figure 1E).

### 2.2. Tumor-Derived SEVs Upregulate PD-L1 Expression in M0-M

It is known that EVs are important players in cell–cell communication, both in physiological and pathological conditions [42]. Recent studies have highlighted the involvement of cancer-derived EVs in tumor immunoescape by inducing phenotypic changes in immune cells [16].

**Figure 2:**
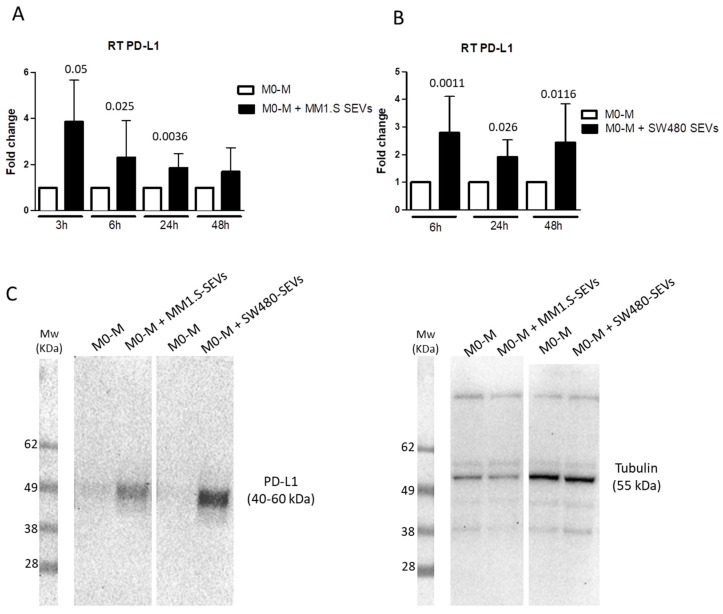
(**A**) RT PCR of PD-L1 mRNA levels in M0-M treated with MM1.S-SEVs for 3, 6, 24, and 48 h. (**B**) RT PCR of PD-L1 mRNA levels in M0-M treated with SW480-SEVs for 6, 24, and 48 h. The reported values are the mean ± standard deviation of three replicates at least. The statistical significance of the differences between the SEV-treated cells and the control for each time point was analyzed using a two-tailed Student’s *t*-test. (**C**) Western Blot analysis of PD-L1 and tubulin proteins in M0-M treated with MM1.S- and SW480-SEVs for 24 h.

To investigate whether tumor-derived SEVs could contribute to creating an immunosuppressive microenvironment, we evaluated the ability of MM-SEVs and CRC-SEVs to induce the expression of PD-L1 in M0-M. As shown in Figure 2A, the treatment with MM1.S-SEVs for 3, 6, and 24 h led to an upregulation of PD-L1 mRNA levels. A similar trend even with different timings (6, 24, and 48 h) occurs in the treatment with SW480-SEVs (Figure 2B). The upregulation of PD-L1 was also confirmed at the protein level after 24 h of treatment with both MM1.S- and SW480-SEVs by Western Blot (Figure 2C and Supplementary Figure S2).

### 2.3. MM- and CRC-SEVs Increase IL-6 Expression and STAT3 Phosphorylation in M0-M

**Figure 3:**
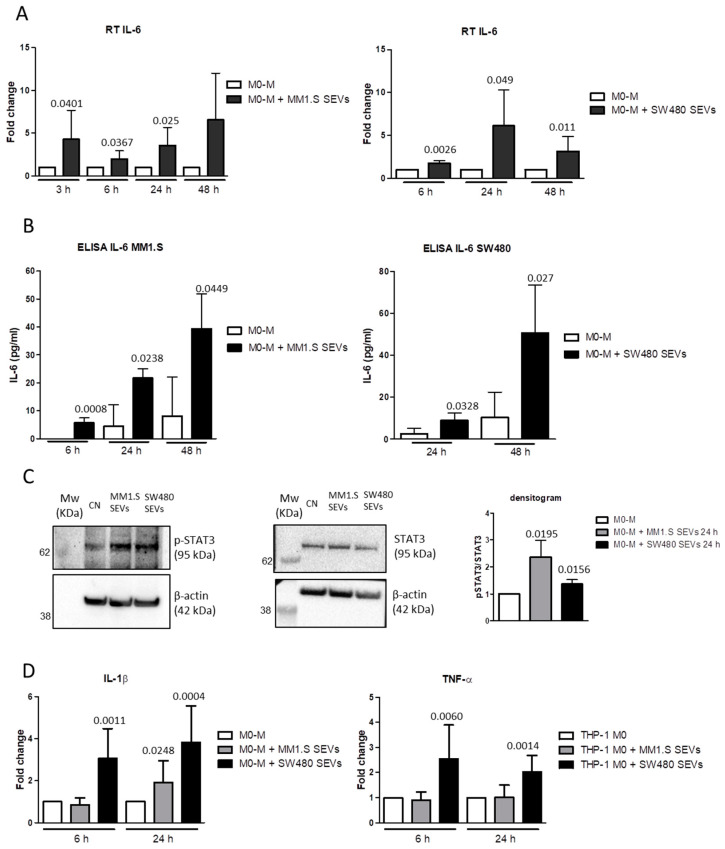
(**A**, left panel) RT PCR of IL-6 mRNA levels in M0-M treated with MM1.S-SEVs for 3, 6, 24, and 48 h. The reported values are the mean ± standard deviation of three replicates at least. (**A**, right panel) RT PCR of IL-6 mRNA levels in M0-M treated with SW480-SEVs for 6, 24, and 48 h. The reported values are the mean ± standard deviation of four replicates at least. (**B**, left panel) ELISA assay of IL-6 released in the conditioned media of M0-M treated with MM1.S-SEVs for 6, 24, and 48 h. The reported values are the mean ± standard deviation of three replicates. (**B**, right panel) ELISA assay of IL-6 released in the conditioned media of M0-M treated with SW480-SEVs for 24 and 48 h. The reported values are the mean ± standard deviation of four replicates. (**C**, left panel) Western Blot analysis of P-STAT3, STAT3, and β-actin proteins in M0-M treated with MM1.S- and SW480-SEVs for 24 h. (**C**, right panel) Densitometric analysis of the Western Blot. The reported values are the mean ± standard deviation of three replicates. (**D**) RT PCR of IL-1β and TNFα mRNA levels in M0-M treated with MM1.S- and SW480-SEVs for 6 and 24 h. The reported values are the mean ± standard deviation of six replicates at least. The statistical significance of the differences between the SEV-treated and untreated M0-M cells for each time point was analyzed using a two-tailed Student’s *t*-test.

The studies performed on several tumor models have demonstrated that the upregulation of PD-L1 is regulated by the IL-6/STAT3 signaling pathway [40,43,44,45,46]. In light of these observations, we evaluated the IL-6 expression and STAT3 activation in M0-M treated with MM- and CRC-SEVs. The results showed that both SEVs induced an increase in IL-6 at the mRNA and protein levels (Figure 3A,B, respectively). In particular, MM1.S-SEVs elicited the upregulation of IL-6 after 3, 6, and 24 h of treatment, while SW480-SEVs elicited their upregulation after 6, 24, and 48 h of treatment. As shown in Figure 3C (and Supplementary Figure S3), we also found that the treatment with MM- and CRC-SEVs after 24 h induced the phosphorylation of STAT3. These observations support the idea that the IL-6 induced by the tumor-derived SEVs released in the extracellular environment through an autocrine loop activates STAT3-mediated signaling, leading to PD-L1 upregulation. Moreover, the CRC-SEVs significantly affected the expression of other inflammatory mediators such as TNF-α and IL-1β, with the latter also upregulated by the MM-SEVs (Figure 3D).

### 2.4. MM and CRC-Derived SEVs Increase NF-kB Expression in M0 Macrophages

**Figure 4:**
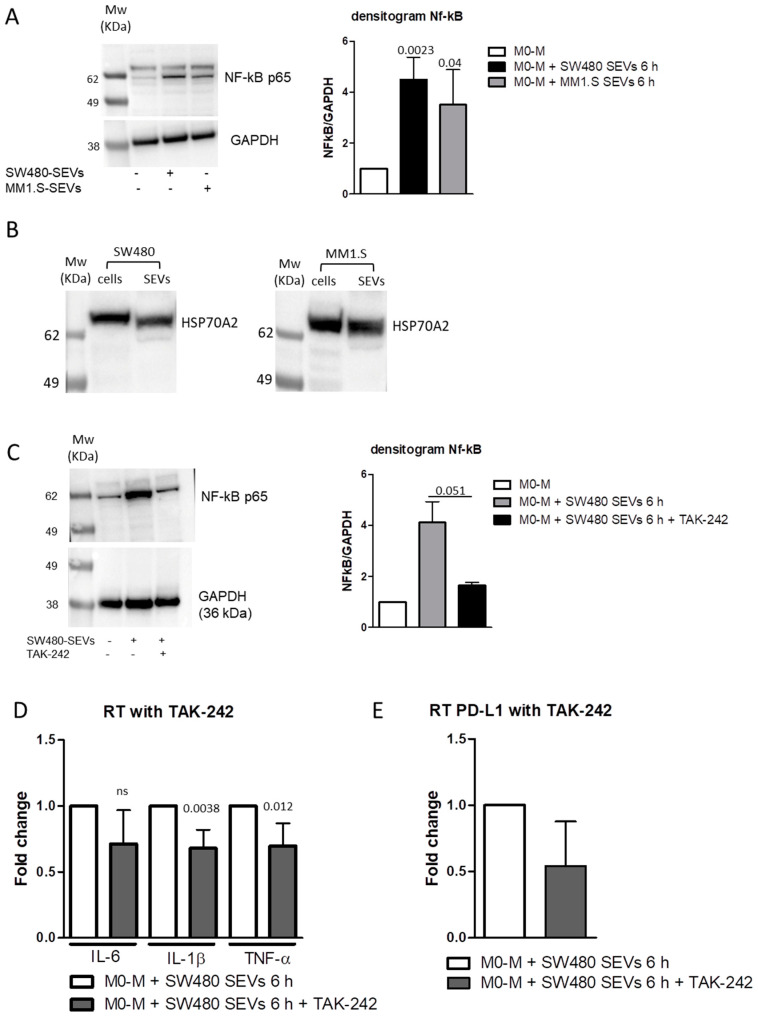
(**A**, left panel) Western Blot analysis of NF-κB and β-actin proteins in M0-M treated with MM1.S- and SW480-SEVs for 6 h. (**A**, right panel) Densitometric analysis of Western Blot. The reported values are the mean ± standard deviation of two replicates. (**B**) Western Blot analysis of HSP70A2 in MM1.S and SW480 cell lysates and derived SEVs. (**C**, left panel) Western Blot analysis of NF-κB and β-actin proteins in M0-M treated with SW480-SEVs and TAK-242 for 6 h. (**C**, right panel) Densitometric analysis of Western Blot. Densitometric analysis of Western Blot. The reported values are the mean ± standard deviation of two replicates. (**D**) RT PCR of IL-6, IL-1β, and TNF-α mRNA levels in M0-M treated with SW480-SEVs and TAK-242 for 6 h. The reported values are the mean ± standard deviation of three replicates at least. (**E**) RT PCR of PD-L1 mRNA levels in M0-M treated with SW480-SEVs and TAK-242 for 6 h. The reported values are the mean ± standard deviation of three replicates. The statistical significance of the differences between the SEV-treated cells and SEV-treated cells plus the inhibitor was analyzed using a two-tailed Student’s *t*-test.

TLR4/MyD88/NF-κB signal pathway plays a key role in regulating the expression of various inflammatory cytokines including IL-6, IL-1β, and TNF-α [46,47], and its activation directly upregulates the PD-L1 expression [39,48]. Thus, we investigated the ability of tumor-derived SEVs to activate this pathway. We observed that, in M0-M, stimulation for 6 h with both MM1.S-SEVs and SW480-SEVs upregulated the expression of NF-κB p65, which represents the active form of NF-κB (Figure 4A and Supplementary Figure S4). Interestingly, we also found the presence of HSP70A2, known to be a ligand of TLR4, in both SEV populations [49,50] (Figure 4B and Supplementary Figure S4). Data reported in the literature described the ability of the SEV-associated HSP70A2 to promote a TLR4-dependent secretion of IL-6 in dendritic cells [49]. According to these, we hypothesize that HSP70A2 can be a molecular mediator of the effects observed after the treatment of M-M0 cells with MM1.S-SEVs and SW480-SEVs. To further define the involvement of the TLR4/NF-κB pathway, we pre-treated M0-M cells with TAK-242, an inhibitor of TLR4, and then, we added MM1.S-SEVs and SW480-SEVs for 6 h. As showed in Figure 4C (and Supplementary Figure S4), we observed that the pre-treatment with TAK-242 was able to inhibit the increase in NF-kB p65 induced by SW480-SEVs (Figure 4D) but not by MM1.S-SEVs (data not shown). According to this data, we observed that the TLR-4 inhibitor significantly blocked the upregulation of pro-inflammatory cytokines IL-1β and TNF-α, induced only by SW480-SEVs (Figure 4D). These results indicate that SEVs with different origins can elicit the same effects acting through multiple pathways. In this case, the TLR4/NF-κB pathway plays a crucial role in mediating the SW480-SEV effects on M0-M, while it has a marginal role on MM1.S-SEV treated cells. Interestingly, we found that the non-significant reduction in IL-6 following treatment with the inhibitor (Figure 4D) corresponds to the absence of a decrease in PD-L1 under the same conditions (Figure 4E); this observation supports the hypothesis of a close correlation between the two factors mediated by an IL-6-dependent autocrine loop.

## 3. Discussion

The aberrant expression of PD-L1 is considered an oncogenic driver since it is involved in tumor immune evasion, limiting the T cell’s response. In this study, we focused on analyzing the effects of colorectal cancer (CRC) and multiple myeloma (MM)-small extracellular vesicles (SEVs) on the modulation of PD-L1 in macrophages. We demonstrated that the expression of PD-L1 on macrophages is increased by CRC- and MM-SEV treatments. To investigate the underlying mechanism, we first analyzed the presence of PD-L1 protein in SEVs. Then, to investigate the possible different biological mechanisms involved in PD-L1 induction, we evaluated whether SEVs may act as a PD-L1 inducer in M0 macrophages.

Our results revealed that tumor SEVs can significantly upregulate the expression of PD-L1 in M0 macrophages at both the mRNA and protein levels. In parallel, we found that SEVs are also able to induce a significant increase in IL-6 expression at both the mRNA and protein levels and to activate the STAT3 signaling pathway; these data suggested an SEV-mediated induction of PD-L1 in M0 macrophages target cells. Data in the literature already showed the role of IL-6 in different steps of tumor development, such as in modeling the immune responses in cancers [33] and in colorectal cancer [35].

Furthermore, IL-6 plays a key role in PD-L1 induction through a STAT3-dependent mechanism in different target cells, such as neutrophils [51] and myeloid cells [34]. Cheng et al. showed that hepatocellular cancer-associated fibroblasts induce PD-L1^+^ neutrophils through the IL-6-STAT3 pathway, which impairs T-cell function, inducing immune suppression through the PD1/PD-L1 signaling pathway [51]. Lamano et al. identified Glioblastoma-Derived IL-6 as a cytokine that is necessary for inducing PD-L1 in Glioblastoma through a STAT3-dependent mechanism; they demonstrated that the inhibition of IL-6 signaling in orthotopic murine glioma models was associated with the decrease in PD-L1 expression and the reduction in of tumor growth and survival [34].

According to the studies present in the literature, our data supported the key role of tumor-SEVs in IL-6 production; in addition, in this study, we correlated the EV-mediated increase in PD-L1 in M0 macrophages to IL-6 release and STAT3 signaling pathway activation.

Furthermore, we focused on other possible and convergent pathways involved in the EV-mediated regulation of PD-L1 expression on M0 macrophages. For example, it was recently found that melanoma-derived EVs can stimulate PD-L1 expression in myeloid cells via TLR4 signaling [39]. Moreover, TLR4 stimulation, besides being involved in the induction of PD-L1, is able to induce myofibroblast/fibroblast-mediated suppression of CD4(+) T cell proliferation, leading to the maintenance of immune-tolerance [48].

Furthermore, data in the literature reported also that NF-κB, a key positive regulator of PD-L1 expression in cancer, is activated by TLR4 [52]. Interestingly, a strong NF-κB activation is observed in the EV-stimulated immortalized myeloid suppressor cell line MSC-2, and PD-L1 upregulation is reduced by the NF-κB inhibitor [39].

Here, we demonstrated that both SEVs are also able to induce a significant upregulation of NF-kB at the protein level, leading to an increase in TNF-α and IL1β expressions; this contributes to the creation of an inflammatory microenvironment able to support and to enhance tumor progression. HSPs are known to bind and induce the activation of TLR signaling pathways [53]. Interestingly, we observed the presence of the HSP70 protein in SEVs hypothesizing the involvement of this protein in TLR4/NF-kB pathway activation in macrophages. However, only CRC-SEVs seem to mediate this stimulation through TLR4 signaling pathway activation since the effects mediated by MM-SEVs were not attenuated by the TLR4 inhibitor (data not shown). The explanation of this phenomenon could be that EVs are packages of a variety of active biomolecules and that PD-L1 expression can be induced by other mediators involved in pathways different from that of TLR4. Further studies are needed to better clarify all of the molecular mechanisms involved.

## 4. Materials and Methods

### 4.1. Cell Cultures

The MM cell line, MM1.S, and the non-metastatic CRC cell line, SW480, were purchased from ATCC (Manassas, VA, USA) and were grown in RPMI 1640 (Euroclone, Pero, Italy) supplemented with 10% fetal bovine serum (FBS; Euroclone), 2 mM L-glutamine (Euroclone), 100 U/mL penicillin, and 100 µg/mL streptomycin (Euroclone). FBS was previously ultracentrifuged to deplete it from extracellular vesicles (extracellular vesicle-free FBS).

### 4.2. THP1-Derived Macrophage Culture

The human monocyte THP-1 cell line was purchased from ATCC (Manassas, VA, USA). The cells were cultured in RPMI-1640 medium (Euroclone) supplemented with 10% FBS, 2 mM L-glutamine, 100 U/mL penicillin, 100 μg/mL streptomycin (Euroclone), and 0.05 mM 2-mercaptoethanol. THP-1 monocytes were differentiated into M0 macrophages (M0-M) as previously described [54,55]. Particularly, the cells were seeded at 1 × 10^5^ cells/mL and incubated at 37 °C with 5% CO_2_ for 48 h in the presence of 50 ng/mL of PMA (Sigma-Aldrich, Saint Luis, MO, USA); then, the conditioned medium with PMA was removed and replaced with fresh medium for 3 days for cell recovery. The macrophages maturation was assessed by flow cytometry. After five days of culture, the cells were harvested and stained for CD68, a marker of mature macrophages, using the antihuman monoclonal antibody (mAb) anti CD68 PE (clone Y1/82A) and its isotype control PE Mouse IgG2b, κ Isotype Ctrl Antibody (clone MPC-11) (Biolegend San Diego, CA, USA). The expression of CD68 in THP-1-derived macrophages was assessed using a FACSARIA flow cytometer (BD biosciences La Jolla, CA, USA). At least 30,000 cells were acquired, and the two-fold increase in mean fluorescence intensity (MFI) of CD68 compared with the isotype control was considered successful maturation.

The THP-1 cells were treated at different time points with small extracellular vesicles derived from the MM1.S and SW480 cell lines. TAK-242 powder, an inhibitor of TLR4, was purchased from Sigma, resuspended in PBS, and stored at −80 °C; before treatment with CRC- and MM-SEVs, the THP-1 cells were treated with TAK-242 (5 μM) for 30 min.

### 4.3. Small Extracellular Vesicles Isolation

Small extracellular vesicles (SEVs) were isolated from the conditioned culture medium of MM1.S and SW480 maintained in the presence of extracellular vesicles-free FBS. The conditioned medium was collected after a culture period of 48 h for MM1.S-derived SEVs, and after 24 h for SW480-derived SEVs. The conditioned medium was subjected to differential centrifugations followed by ultracentrifugation as previously described [56,57]. Briefly, the conditioned culture medium was centrifuged for 5 min at 300× *g*, 15 min at 3000× *g*, and 30 min at 10,000× *g*; the supernatant was then ultracentrifuged for 105 min at 100,000× *g* in a Type 70 Ti, fixed angle rotor. The pellets were suspended in PBS or lysis buffer, and the SEV protein content was determined by the Bradford assay.

### 4.4. Trasmission Electron Microscopy (TEM)

The isolated SEVs were prepared for transmission electron microscopy studies using negative staining. A 5 μL aliquot of SEV suspension was deposited onto 200 mesh carbon-coated EM grids. After washing, the samples were fixed for 5 min in 1% glutaraldehyde and then negatively stained with 2% aqueous solution of phosphotungstic acid. The grids were viewed in a JEM 1400 Plus electron microscope (JEOL Ltd, Tokyo, Japan) operating at 80 kV.

### 4.5. Internalization of SEVs by M0 Macrophages

MM1.S- and SW480-derived SEVs (MM.1S-SEVs and SW480-SEVs, respectively) were labeled with PKH26 Red Fluorescent Cell Linker Kits (Merck KGaA, Darmstadt, Germany) following the datasheet information. Briefly, SEVs were incubated with PKH26 dye for 15 min at room temperature, washed twice in PBS, and resuspended in the growth medium. The labeled SEVs were incubated with THP-1 cells for 3 and 6 h at 37 °C with 5% CO_2_. After incubation, the cells were fixed with PFA 4%, permeabilized with 0.1% TritonX-100, and stained with Actin Green (Molecular Probes, Life Technologies, Carlsbad, CA, USA, 1 drop/mL of PBS) that binds actin with high affinity, and the nuclei were stained with Hoechst (Molecular Probes, Life Technologies, Carlsbad, CA, USA, diluition 1:1000). The samples were analyzed by confocal microscopy (Nikon A1, Amsterdam, Netherlands).

### 4.6. MTT (3-[4,5-Dimethylthiazol-2-yl]-2,5 Diphenyl Tetrazolium Bromide) Assay

Cell viability was determined by an MTT assay as previously described [58]. The THP-1 cells were seeded in triplicate in 48-well plates; 24 h post-seeding, the cells were treated with MM1.S (50 µg/mL) and SW480 (20 µg/mL)-derived SEVs for 24 and 48 h. The absorbance was measured by an ELISA reader at 540 nm (Microplate Reader, BioTek, Winooski, VT, USA). Values are expressed as a percentage of cell growth versus that in the control (untreated cells).

### 4.7. Western Blotting

Total proteins from the THP-1 cells treated with MM1.S- and SW480-derived SEVs and from MM1.S- and SW480-derived SEVs were isolated and analyzed by SDS-PAGE followed by Western Blotting. The primary antibodies used in the experiments were as follows: anti-PD-L1 antibody (Abcam, Cambridge, UK, ab213524, diluition 1:1000; negative and positive controls of the antibody are included in Supplementary Figure S5), anti-pSTAT3 (R&D System, AF4607-SP, diluition 1:500), anti-STAT3 (Novus Biologicals, Denver, CO, USA, NBP2-24463, diluition 1:1000), anti-HSP70 (Novus Biologicals, NB600-1469, diluition 1:1000), anti-HSC70 (Santa Cruz, Dallas, TX, USA, sc-7298, diluition 1:500), anti-Calnexin (Santa Cruz, sc-23954, diluition 1:500), anti-Tubulin (Santa Cruz, sc-398103, diluition 1:1000), anti-β Actin (Santa Cruz, sc-81178, diluition 1:1000), anti-GAPDH (Santa Cruz, sc-47724, diluition 1:1000), and anti-NF-kB (Novus, NB100-2176, diluition 1:500). After overnight incubation with the primary antibodies at 4 °C, the membranes were incubated with HRP-conjugated secondary antibody (Thermo Fisher Scientific, Cambridge, MA, USA) for 1 h at 4 °C; the chemiluminescent signal was detected by Chemidoc (Biorad, Milan, Italy).

### 4.8. Real-Time PCR

The THP-1 cells were seeded in 12 well-plates at 1 × 10^5^ cells/mL and differentiated in M0 macrophages, as described above; the cells were then treated with MM1.S (50 µg/mL) and SW480 (20 µg/mL)-derived SEVs for 6 and 24 h. At the end of the treatments, total RNA was extracted using Illustra^TM^ RNA spin mini-RNA isolation Kit (GE Healthcare, Little Chalfont, Buckinghamshire, UK). The RNA was reverse transcribed to cDNA using the High-Capacity cDNA Reverse Transcription kit (Applied Biosystems, Foster City, CA, USA). Then, the cDNA was subjected to quantitative real-time reverse transcriptase-polymerase chain reaction (RT-PCR) analysis. The sequences of the primers used were indicated in Table 1:

Real-time PCR was performed using Step OneTM Real-time PCR System Thermal Cycling Block (Applied Biosystem, Waltham, MA, USA) in a 20 μL reaction containing 300 nM of each primer, 2 μL of template cDNA, and 18 μL of 2X SYBR Green I Master Mix. The PCR was run at 95 °C for 20 s followed by 40 cycles of 95 °C for 3 s and 60 °C for 30 s. GAPDH was used as the endogenous control. Relative changes in gene expression between control and treated samples were determined using the ΔΔCt method.

### 4.9. Enzyme-Linked ImmunoSorbent Assay (ELISA)

The amount of IL-6 in the culture medium of THP-1 cells treated with SEVs was determined using a human IL-6 ELISA kit (R&D Systems, Inc, Minneapolis, MN, USA) IL-6 protein levels were also analyzed in MM1.S- and SW480-derived SEV protein lysates. The ELISA assay was performed according to the manufacturer’s instructions.

### 4.10. Statistical Analysis

Data are reported as mean ± standard deviation (SD) of three or more biological replicates. Statistical analysis was performed using GraphPad Prism software (GraphPad Software, Inc, La Jolla, CA, USA). The statistical significance of the differences was analyzed using Student’s *t*-test. A *p*-value ≤ 0.05 was considered significant.

## 5. Conclusions

**Figure 5:**
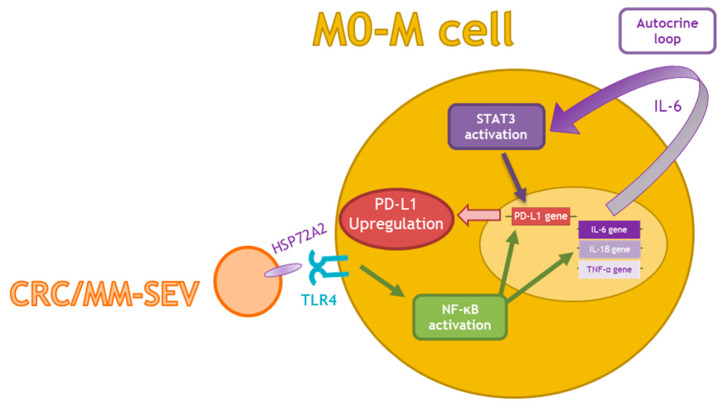
Schematic representation of the proposed model: MM- and CRC-SEVs carry HSP70A2, which can bind TLR4 and in turn activate NF-κB. NF-κB enhances the transcription of both PD-L1 and pro-inflammatory cytokines, such as IL-6, IL-1β, and TNF-α. The increase in IL-6 turns into an autocrine loop that promotes the activation of STAT3, thus leading to an increase in PD-L1 expression.

In conclusion, the results of the present study indicate that EVs from both CRC and MM cells modulate pro-inflammatory cytokines and PD-L1 expression in M0 macrophages through IL-6/STAT3 and the HSP72/TRL4/Nf-kB axes (Figure 5). These data highlighted a new role of tumor derived EVs in promoting macrophage differentiation and tumor immunoescape. Deeper dissections of the identified mechanism may allow for developing new therapeutic strategies to prevent cancer development.

## Figures and Tables

**Table 1:** List of primers used for Real-Time PCR.

Primers	Forward	Reverse
GAPDH	ATGGGGAAGGTGAAGGTCG	GGGTCATTGATGGCAACAATAT
PD-L1	TCACGGTTCCCAAGGACCTA	AGGTCTTCCTCTCCATGCAC
IL-6	GGTACATCCTCGACGGCATCT	GTGCCTCTTTGCTGCTTTCAC
IL-1β	ACAGATGAAGTGCTCCTTCCA	GTCGGAGATTCGTAGCTGGAT
TNF-α	CCAGGCAGTCAGATCATCTTCTC	AGCTGGTTATCTCTCAGCTCCAC

## Data Availability

Data are available from the corresponding authors upon reasonable request.

## Supplementary Materials

The following are available online at https://www.mdpi.com/article/10.3390/ijms222212118/s1.
