# Supplementary material for: Tumor-Derived Small Extracellular Vesicles Induce Pro-Inflammatory Cytokine Expression and PD-L1 Regulation in M0 Macrophages via IL-6/STAT3 and TLR4 Signaling Pathways
Source: Int J Mol Sci. 2021 Nov 9;22(22):12118. doi: 10.3390/ijms222212118 (PMC8621495; doi:10.3390/ijms222212118)
Supplement: Supplementary file 1 [file ijms-22-12118-s001.zip › ijms-1431495-supplementary.pdf]

International Journal of
Molecular Sciences

MDPI

Supplementary Figures

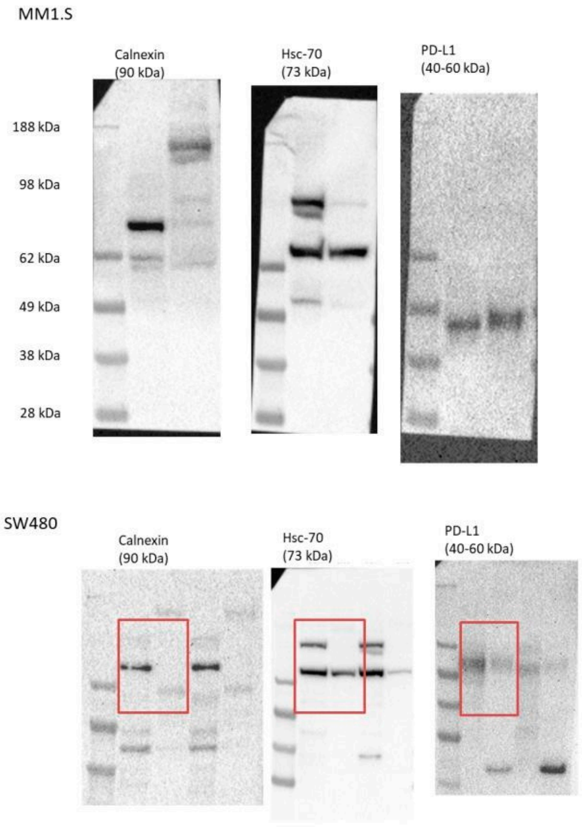

Supplementary Figure S1. Uncropped images of western blot in Figure 1C

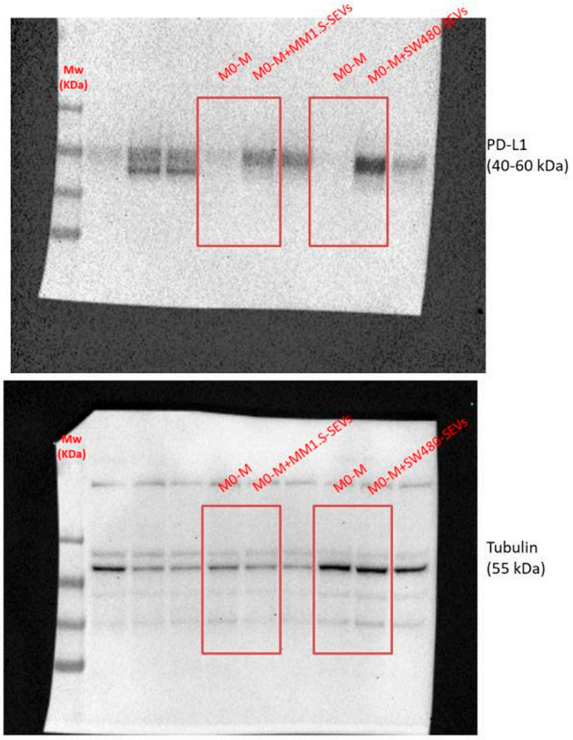

Supplementary Figure S2. Uncropped images of western blot in Figure 2C

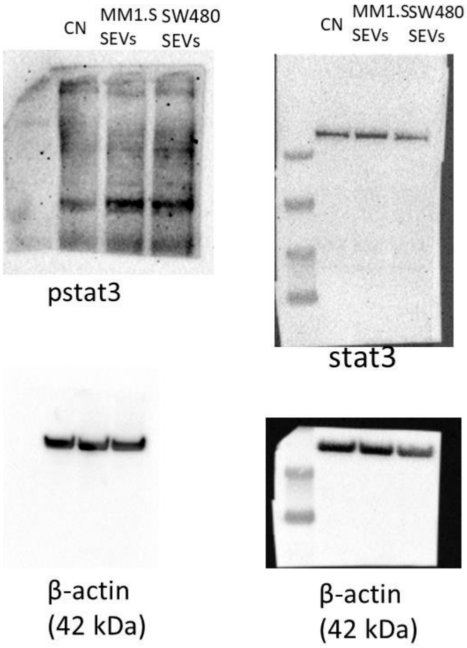

Supplementary Figure S3. Uncropped images of western blot in Figure 3C

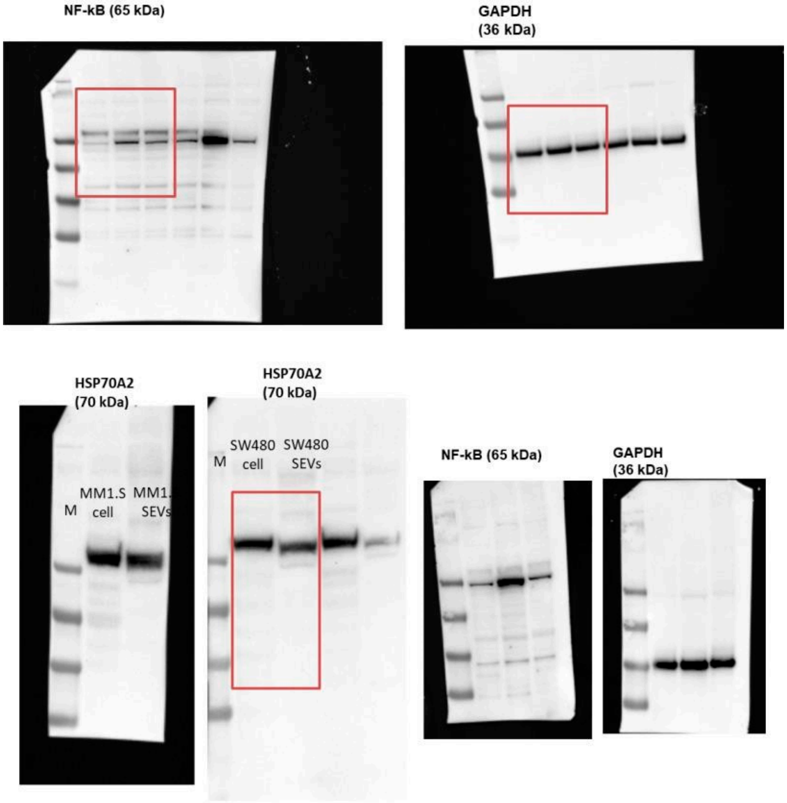

Supplementary Figure S4. Uncropped images of western blot in Figure 4 A-B-C

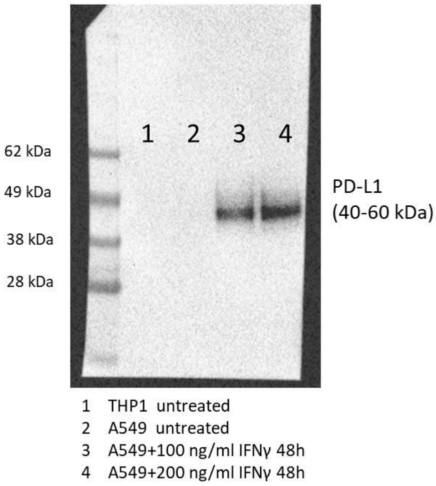

Supplementary Figure S5. Negative (THP-1 and A549 untreated) and Positive (A549 treated for 48h with 100 and 200 ng/ml of IFN-γ) controls of PD-L1 antibody.
